# Mortality in adolescents receiving special education and social care provision: A population-level cohort study in England

**DOI:** 10.23889/ijpds.v9i5.2684

**Published:** 2024-09-18

**Authors:** Isobel Ward, Matthew Jay, Joseph Ward, Ruth Gilbert, Ania Zylbersztejn

**Affiliations:** 1 UCL Great Ormond Street Institute of Child Health

## Background

Over a third of children in England receive either special educational need (SEN) provision/children’s social care (CSC) support at least once during school. We aimed to describe all-cause and adversity-related mortality for adolescents who received SEN or CSC provision in England.

## Approach

We used linked data from hospital admissions, death registrations, social care and education in the ECHILD database to follow all adolescents in state schools from age 13-years between 2009/10-2014/15 until death or 2020. Adolescents were grouped according to provision ever recorded before age 14: None, SEN only, CSC only or CSC and SEN. We estimated all-cause and adversity-related mortality in children with SEN/CSC/both compared to peers.

## Results

The cohort comprised 3,381,502 children (51% boys), of whom 30.1% (n=1,017,670, 62% boys) received SEN only, 4.2% (n=143,430, 36% boys) received CSC only and 8.7% (n=293,310, 57% boys) received SEN and CSC. In ten-year follow up, 6,590 deaths occurred (66% in boys). Mortality rates were 5.4 times higher for adolescents receiving both SEN and CSC, 2.4 times higher for CSC only, and 1.7 times higher for SEN only compared to peers. Rates of adversity-related deaths were highest for boys receiving SEN and CSC compared to peers.

## Conclusions & Implications

8-in-1000 adolescents who received both SEN and CSC before age 13 died by age 23 years, far more often than their peers. Policies need to consider support for young people with additional educational and social care needs as they leave school and transition to adulthood.

